# Meta-analysis of emotion recognition deficits in major depressive disorder

**DOI:** 10.1017/S0033291714002591

**Published:** 2014-11-14

**Authors:** M. N. Dalili, I. S. Penton-Voak, C. J. Harmer, M. R. Munafò

**Affiliations:** 1 School of Experimental Psychology, University of Bristol, UK; 2 MRC Integrative Epidemiology Unit (IEU), University of Bristol, UK; 3 Department of Psychiatry, University of Oxford, UK; 4 UK Centre for Tobacco Control Studies, University of Bristol, UK

**Keywords:** Emotion recognition, major depressive disorder, meta-analysis

## Abstract

**Background.:**

Many studies have explored associations between depression and facial emotion recognition (ER). However, these studies have used various paradigms and multiple stimulus sets, rendering comparisons difficult. Few studies have attempted to determine the magnitude of any effect and whether studies are properly powered to detect it. We conducted a meta-analysis to synthesize the findings across studies on ER in depressed individuals compared to controls.

**Method.:**

Studies of ER that included depressed and control samples and published before June 2013 were identified in PubMed and Web of Science. Studies using schematic faces, neuroimaging studies and drug treatment studies were excluded.

**Results.:**

Meta-analysis of *k* = 22 independent samples indicated impaired recognition of emotion [*k* = 22, *g* = −0.16, 95% confidence interval (CI) −0.25 to −0.07, *p* < 0.001]. Critically, this was observed for anger, disgust, fear, happiness and surprise (*k*'s = 7–22, *g*'s = −0.42 to −0.17, *p*'s < 0.08), but not sadness (*k* = 21, *g* = −0.09, 95% CI −0.23 to +0.06, *p* = 0.23). Study-level characteristics did not appear to be associated with the observed effect. Power analysis indicated that a sample of approximately 615 cases and 615 controls would be required to detect this association with 80% power at an alpha level of 0.05.

**Conclusions.:**

These findings suggest that the ER impairment reported in the depression literature exists across all basic emotions except sadness. The effect size, however, is small, and previous studies have been underpowered.

## Introduction

The perception of emotion from non-verbal cues is crucial to human social interaction. Many psychological disorders are characterized by deficits or biases in facial emotion recognition (ER), including schizophrenia (Addington *et al.* 2006), alcoholism (Philippot *et al.* 1999), autism (Celani *et al.* 1999), anxiety (Button *et al.* 2013*a*), bipolar disorder (Derntl *et al.* 2009), and depression (Rubinow & Post, 1992).

Affective disorders affect 21 million people in Europe alone and account for nearly half of the costs of all mental disorders (Andlin-Sobocki *et al.* 2005). Understanding the role of ER is especially relevant to depression, as the impaired recognition of emotion has been associated with decreased satisfaction, support, and well-being of interpersonal relationships (Carton *et al.* 1999). Critically, poor interpersonal relationships have been proposed as an important factor in both the aetiology and maintenance of depression (Finch & Zautra, 1992, Platt *et al.* 2013), and impaired ER may contribute to the interpersonal difficulties and avoidance seen in depression (Persad & Polivy, 1993). Since deficits in ER may contribute to the maintenance of depressive symptoms, investigating this relationship has important implications for existing cognitive behavioural interventions and the development of novel interventions.

Many studies have attempted to investigate the relationship between ER and depression over the last 30 years (see Bourke *et al.* 2010 for a review). However, these have used various paradigms and stimulus sets, thus making the comparison of results across studies difficult. Two recent meta-analyses were conducted to investigate the association between major depressive disorder (MDD) and ER. Demenescu *et al.* (2010) examined eight studies and found that ER in depressed adults was moderately impaired compared to controls. Given the small number of included studies, analyses stratified by emotion and analyses of study-level design characteristics were not conducted. Similarly, Kohler *et al.* (2011) identified a moderate deficit in ER in a meta-analysis of 51 studies of emotion identification or discrimination in bipolar (31 studies) or unipolar (20 studies) depressed patients compared to controls. Notably, impairment did not differ between diagnostic groups, and analyses of all six basic emotions revealed small to moderate deficits across both patient groups. However, data on specific emotions were limited, so it was difficult to determine with certainty whether the nature and strength of the deficit differed by emotion. There was also some evidence suggesting that symptom severity was associated with a greater deficit in ER. Furthermore, demographic characteristics such as old age, sex (females), and higher levels of education (in cases) were also shown to be positively associated with ER performance.

The results from these meta-analyses are inconclusive regarding whether the ER deficit in depression is general or specific to the recognition of one or more emotions. The discovery of a specific ER deficit in depression would have important implications for treatment, allowing clinicians to target the treatment of impairments more effectively. Some researchers have proposed that there is a unique relationship between MDD and the recognition of happiness, suggesting that the recognition of happiness is specifically impaired while the recognition of sadness is spared or enhanced (Gur *et al.* 1992; Bourke *et al.* 2010). Similarly, while studies have demonstrated that some antidepressant pharmacotherapies modify the recognition of emotion (Harmer *et al.* 2011, 2013), meta-analyses thus far have not considered the effects of current medication on ER in depressed individuals.

The purpose of this meta-analysis was therefore to extend our understanding of the relationship between ER deficits and MDD. We did this by comparing studies across several different methodologies, paradigms, and design-level characteristics, including stimulus sets, presentation times, and response options. This included stratifying our analyses by medication status (i.e. medicated or unmedicated) in order to investigate the effects of antidepressants on this relationship. In addition to investigating a general deficit of ER, we further stratified our analyses by all six basic emotions in order to investigate specific deficits. In the interest of reducing the moderate levels of heterogeneity detected in the previous meta-analyses, we only included studies using human facial emotional expression stimuli. Finally, we also calculated the statistical power of each study included in our analysis to detect the effect size indicated by the meta-analysis, and tested for possible publication bias. This meta-analysis extends our understanding of the relationship between ER abilities, and provides a more accurate estimate of the real magnitude of the effect of depression on ER deficits in studies using photorealistic stimuli.

## Method

### Study inclusion/exclusion criteria

Eligibility criteria for study inclusion were as follows: (1) studies were required to have both a clinical sample with a diagnosis of MDD and a control sample; (2) studies were required to have assessed the accuracy of ER; and (3) studies were required to have used stimuli comprising of human facial emotional expressions. Studies using schematic or artistically rendered faces, neuroimaging studies and studies that included experimental administration of drug treatments were excluded. Studies that recruited participants with a diagnosis of both MDD and bipolar disorder were retained.

### Search strategy

We performed a search on two databases: PubMed and Web of Science. These databases were searched from the first date available in each database up to 1 June 2013, using the inclusion terms ‘depression’, ‘MDD’, ‘emotion*’, ‘recognition’, ‘perception’ and the exclusion term ‘administration’. After articles had been collected, bibliographies were then searched for additional references.

### Data extraction

For each study, the following data were extracted: (1) author(s) and year of publication; (2) data [mean and standard deviation (s.d.) of ER accuracy scores, number of participants, mean age and male/female ratio] and (3) study design characteristics. Study design was coded (where possible/applicable) for: stimulus emotion (anger, disgust, fear, happiness, sadness, surprise), case status (MDD no co-morbidity, MDD co-morbidity, MDD+bipolar disorder), control status (matched, unmatched), treatment status (medicated, unmedicated), diagnostic criteria [Diagnostic and Statistical Manual of Mental Disorders (DSM)/Research Diagnostic Criteria (RDC), International Classification of Diseases (ICD)], stimuli (Ekman & Friesen, Other), use of morphed stimuli (no, yes), stimulus type (dynamic, static), presentation time (<500, >500, 500 ms, self-paced), and response option [two alternative forced choice (AFC), six AFC, other]. We also rated the quality of all included studies using eight items adapted from the Newcastle–Ottawa Scale, a measure for assessing the quality of non-randomized studies in meta-analyses (Wells *et al.* 2000). Studies were rated on the selection of study groups, the comparability of those groups, and the ascertainment of the outcome of interest.

### Data analysis

Effect sizes (Hedges' *g*) were calculated for the comparison of cases *v.* controls on ER accuracy for each emotion reported within each individual study. Hedges' *g* is a measure of standardized mean difference, similar to Cohen's *d* but including a correction for small sample size. Conventionally, a small effect size is defined as 0.20, a medium effect size as 0.50 and a large effect size as 0.80 (Cohen, 1988).

Data were analysed within a random-effects framework, with *g* values pooled using DerSimonian & Laird (1986) methods. A random-effects framework assumes that between-study variation is due to both chance or random variation and an individual study effect, and provides an estimate of the range of likely effect sizes across the populations sampled by individual studies. Random-effects models are more conservative than fixed-effects models and generate a wider confidence interval (CI), but give similar results under conditions of low between-study heterogeneity. The significance of the pooled *g* values was determined using a *Z* test. Between-study heterogeneity was estimated using the *I*^2^ statistic. Conventionally, values of 25%, 50% and 75% represent the upper thresholds for low, moderate and high heterogeneity, respectively.

Small study bias, which may reflect publication bias against null results, was assessed using Egger's test (Egger *et al.* 1997). We also conducted a series of stratified analyses and meta-regression analyses to assess the impact of various study design characteristics. The analyses were conducted using the Comprehensive Meta-Analysis v. 2 statistical software package (Biostat, USA). Exact *p* values are reported throughout.

## Results

### Description of studies

Our search strategy across both databases initially identified 728 articles. Of these, 66 articles were identified as duplicates and were removed. Of the remaining 662 articles, we were able to exclude 624 articles because they did not meet our inclusion criteria. A further 16 articles were excluded because they did not report the data required to enable inclusion in our meta-analysis, and attempts to contact the study authors to acquire these were unsuccessful.

**Fig. 1.**
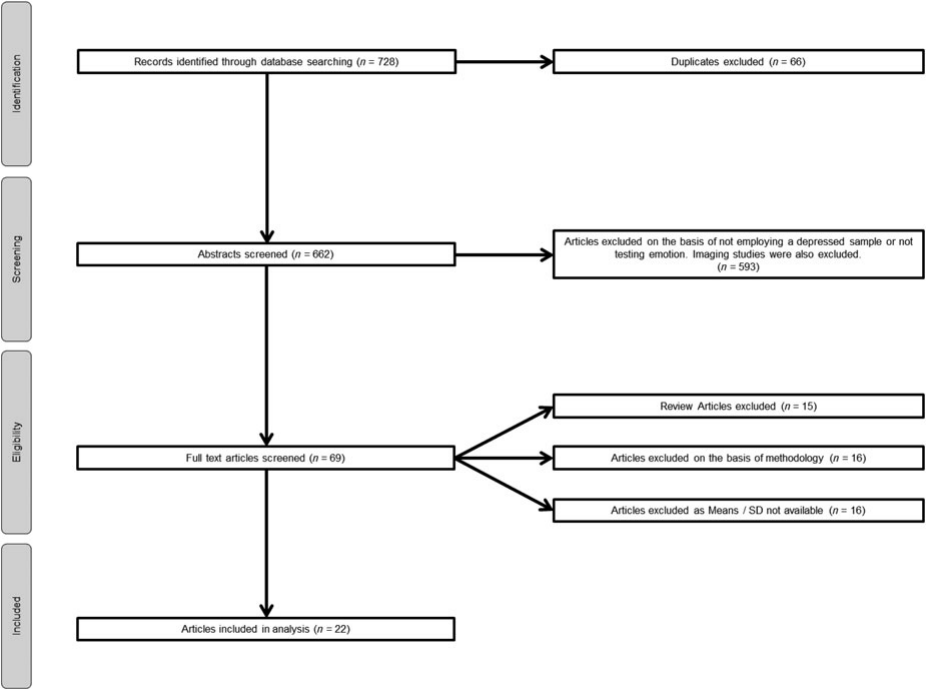
Flow diagram of search results.

A total of 22 studies published between 1992 and 2012 met inclusion criteria and were included in our meta-analysis. A flow chart describing this process is shown in Fig. 1. Characteristics of these studies are described in Table 1.

**Table 1.** Characteristics of included studies

Author	Year	Anger	Disgust	Fear	Happiness	Sadness	Surprise	*N* (cases)	*N* (controls)	Effect (g)	Power (%)	Treatment	Cases	Controls	Diagnosis	Stimuli	Stimuli morphed	Stimulus type	Presentation time	Response option	Age, yr (cases)	Age, yr (controls)	% female (cases)	% female (controls)
Anderson	2011	x	x	x	x	x	x	30	101	−0.02	13	Medicated	Co-morbidity	Unmatched	DSM/RDC	Ekman/Friesen	Yes	Static	>500 ms	Other	39	30	73	57
Arteche	2011				x	x		21	34	−0.40	9	Medicated	Co-morbidity	Unmatched	DSM/RDC	Other	Yes	Dynamic	500 ms	2 AFC	32	34	100	100
Bediou	2005	x	x	x	x			20	20	−0.12	8	Medicated	No co-morbidity	Unmatched	DSM/RDC	Other	Yes	Static	<500 ms	Other	39	26	35	35
Derntl	2012	x	x	x	x	x		24	24	−0.21	9	Medicated	No Co-morbidity	Matched	DSM/RDC	Other	No	Static	>500 ms	2 AFC	41	40	50	50
Douglas	2010	x	x	x	x	x		68	50	−0.30	15	Medicated	Bipolar disorder	Unmatched	DSM/RDC	Ekman/Friesen	Yes	Static	500 ms	6 AFC	40	39	59	63
Gaebel	1992	x	x	x	x	x	x	21	15	−0.02	8	Medicated	No co-morbidity	Unmatched	DSM/RDC	Ekman/Friesen	No	Static	>500 ms	Other	39	31	43	40
Gollan	2010	x	x	x	x	x	x	44	44	−0.09	12	Drug-Free	No co-morbidity	Unmatched	DSM/RDC	Ekman/Friesen	Yes	Static	500 ms	6 AFC	28	31	57	68
Gur	1992				x	x		14	14	−1.11	7	Medicated	Bipolar disorder	Matched	DSM/RDC	Other	No	Static	>500 ms	Other	45	37	86	86
Joormann	2006	x		x	x	x		21	25	−0.16	9	Medicated	Co-morbidity	Unmatched	DSM/RDC	Ekman/Friesen	Yes	Dynamic	500 ms	Other	34	32	86	68
Kan	2004	x	x	x	x	x	x	16	20	−0.10	8	Medicated	No co-morbidity	Unmatched	DSM/RDC	Other	No	Dynamic	>500 ms	6 AFC	51	59	44	50
Langenecker	2007	x		x	x	x		200	71	−0.14	23	Medicated	Co-morbidity	Unmatched	DSM/RDC	Ekman/Friesen	No	Static	<500 ms	Other	35	25	68	57
Leppanen	2004				x	x		18	18	−0.22	8	Medicated	No co-morbidity	Matched	ICD	Ekman/Friesen	No	Static	<500 ms	Other	45	45	61	61
Mah	2010			x	x	x		11	11	−0.11	7	Drug-Free	No co-morbidity	Unmatched	DSM/RDC	Other	No	Static	Self-paced	Other	73	75	64	73
Mendlewicz	2005	x	x	x	x	x		21	32	−0.62	9	Medicated	No co-morbidity	Unmatched	DSM/RDC	Other	Yes	Static	Self-paced	Other	17	21	100	100
Milders	2010	x	x	x	x	x		19	25	−0.06	8	Medicated	Co-morbidity	Matched	ICD	Ekman/Friesen	Yes	Static	500 ms	6 AFC	46	48	58	72
Naranjo	2011	x		x	x	x		23	23	−0.54	9	Medicated	No co-morbidity	Matched	DSM/RDC	Other	No	Static	Self-paced	Other	41	40	78	78
Persad	1993	x	x	x	x	x	x	16	16	−0.51	8	Medicated	No co-morbidity	Unmatched	DSM/RDC	Ekman/Friesen	No	Static	Self-paced	Other	n/a	n/a	100	100
Schaefer	2010	x	x	x	x	x	x	34	24	−0.21	10	Drug-Free	No co-morbidity	Unmatched	DSM/RDC	Ekman/Friesen	Yes	Dynamic	<500 ms	6 AFC	45	45	44	50
Schepman	2012	x		x	x	x		29	37	−0.04	10	Medicated	Co-morbidity	Matched	DSM/RDC	Other	Yes	Static	>500 ms	Other	16	15	66	62
Sprengelmeyer	2011	x	x	x	x	x	x	10	45	−0.40	8	Medicated	Co-morbidity	Unmatched	DSM/RDC	Ekman/Friesen	Both	Static	>500 ms	6 AFC	50	51	70	70
Vederman	2012	x		x	x	x		78	66	−0.01	17	Medicated	No co-morbidity	Unmatched	DSM/RDC	Ekman/Friesen	No	Static	<500 ms	Other	39	37	69	64
Wright	2009	x		x	x	x		239	128	−0.17	34	Medicated	Co-morbidity	Unmatched	DSM/RDC	Ekman/Friesen	No	Static	<500 ms	Other	26	25	71	47

DSM, Diagnostic and Statistical Manual of Mental Disorders; RDC, Research Diagnostic Criteria; ICD, International Classification of Diseases; AFC, alternative forced choice.

### Quality of included studies

The eight items we used to assess the quality of our included studies consisted of four items related to study group selection, two items related to the comparability of groups and two items related to how the studies ascertained the outcome of interest. Each study scored 1 point for each item if the criterion was met. Most studies included in our meta-analysis adequately described the selection of study groups as only three studies scored <3 out of a possible 4 points on these items. Most studies failed to offer sufficient information regarding the comparability of study groups as only six studies scored points on both items while three studies earned only 1 point. All studies met criteria regarding the ascertainment of the outcome of interest, scoring a point for both items.

### ER in MDD

Meta-analysis (*k* = 22) indicated strong evidence of a deficit in ER among cases compared to controls (*g* = −0.16, 95% CI −0.25 to −0.07, *p* < 0.001) with negligible between-study heterogeneity (*I*^2^ = 0%). Stratified analyses across the six primary emotions indicated a deficit in ER for anger, disgust, fear, happiness and surprise (*k*'s = 7–22, *g*'s = −0.42 to −0.17, *p*'s < 0.08), but not sadness (*k* = 21, *g* = −0.09, 95% CI −0.23 to +0.06, *p* = 0.23). Sensitivity analysis indicated that no single study disproportionately contributed to these results. These results are presented in Table 2.

**Table 2.** Meta-analysis of emotion recognition in major depressive disorder by emotion

			95% CI				
	*k*	*G*	Lower	Upper	*p*	*I*^2^ (%)	*p*_Egger_
All studies	22	−0.162	−0.250	−0.074	<0.001	0	0.003
Emotion							
Anger	16	−0.220	−0.376	−0.062	0.006	57	0.003
Disgust	11	−0.420	−0.646	−0.195	<0.001	53	0.69
Fear	17	−0.248	−0.372	−0.123	<0.001	35	0.50
Happiness	22	−0.167	−0.255	−0.080	<0.001	0	0.38
Sadness	21	−0.088	−0.234	+0.057	0.23	56	0.028
Surprise	7	−0.170	−0.358	+0.018	0.076	0	0.29

CI, Confidence interval.

### Impact of study-level design characteristics

Stratified analyses indicated no evidence that any study-level design characteristics altered the deficit in ER among cases compared to controls (*p*'s ⩾ 0.11). In all cases, between-study heterogeneity was moderate to negligible (*I*^2^ ⩽ 55%), with the exception of the two studies in the MDD+bipolar disorder stratum (*I*^2^ = 70%). These results are presented in Table 3. Meta-regression indicated a positive association between year of publication and effect-size estimate (*p* = 0.029).

**Table 3.** Meta-analysis of emotion recognition in major depressive disorder (MDD) by study design characteristics

			95% CI				
	*k*	*g*	Lower	Upper	*p*	*I*^2^ (%)	*p*_diff_
Cases^a^							
MDD no co-morbidity	12	−0.210	−0.363	−0.057	0.007	0	0.27
MDD co-morbidity	8	−0.104	−0.218	+0.009	0.071	0	
MDD + bipolar disorder	2	−0.638	−1.452	+0.176	0.12	70	
Controls							
Matched	6	−0.247	−0.546	+0.052	0.11	55	0.71
Unmatched	16	−0.187	−0.291	−0.084	<0.001	0	
Medication^b^							
Medicated	19	−0.170	−0.265	−0.075	<0.001	4	0.83
Unmedicated	3	−0.134	−0.440	+0.172	0.39	0	
Diagnostic criteria							
DSM/RDC	20	−0.165	−0.256	−0.074	<0.001	0	0.81
ICD	2	−0.119	−0.476	+0.238	0.51	0	
Stimuli							
Ekman & Friesen	13	−0.163	−0.269	−0.056	0.003	0	0.34
Other	9	−0.295	−0.544	−0.045	0.021	46	
Stimuli morphed^c^							
No	12	−0.197	−0.320	−0.075	0.002	0	0.43
Yes	11	−0.126	−0.253	+0.001	0.051	0	
Stimulus type							
Dynamic	4	−0.235	−0.521	+0.052	0.11	0	0.64
Static	18	−0.162	−0.259	−0.064	0.001	6	
Presentation time							
Self-paced	4	−0.504	−0.830	−0.177	0.003	0	0.26
>500 ms	7	−0.182	−0.413	+0.050	0.13	42	
500 ms	5	−0.196	−0.397	+0.005	0.056	0	
<500 ms	6	−0.140	−0.277	−0.002	0.046	0	
Response option							
2 AFC	2	−0.313	−0.708	+0.082	0.12	0	0.11
4 AFC	7	−0.074	−0.191	+0.044	0.22	0	
6 AFC	6	−0.201	−0.388	−0.013	0.036	0	
Other	7	−0.162	−0.568	−0.144	0.001	26	

CI, Confidence interval, DSM, Diagnostic and Statistical Manual of Mental Disorders; RDC, Research Diagnostic Criteria; ICD, International Classification of Diseases; AFC, alternative forced choice.

a One study with cases classified as MDD+BP included participants diagnosed with co-morbid disorders (Douglas & Porter, 2010).

b Studies classified as ‘medicated’ include those where only a proportion of participants were medicated (Gur *et al.* 1992; Kan *et al.* 2004; Bediou *et al.* 2005; Mendlewicz *et al.* 2005; Joormann & Gotlib, 2006; Langenecker *et al.* 2007; Wright *et al.* 2009; Douglas & Porter, 2010; Milders *et al.* 2010; Anderson *et al.* 2011; Arteche *et al.* 2011; Derntl *et al.* 2012; Vederman *et al.* 2012).

c One study used both morphed and unmorphed stimuli in two separate tasks, and contributed to each stratum of this analysis (Sprengelmeyer *et al.* 2011).

### Impact of medication status on recognition of happiness and sadness

Given evidence from human psychopharmacology studies indicating that antidepressants modify ER (Harmer *et al.* 2011, 2013), we examined the impact of medication status on the recognition of happiness and sadness. The pattern of results described did not differ by medication status for either the recognition of happiness (*p* = 0.84) or sadness (*p* = 0.65). Notably, only three studies included in our analysis tested unmedicated cases compared to 19 studies assessing recognition of happiness in medicated samples and 18 assessing recognition of sadness. These results are presented in Table 4.

**Table 4.** Meta-analysis of happiness and sadness recognition in major depressive disorder by medication status

			95% CI				
	*k*	*g*	Lower	Upper	*p*	*I*^2^ (%)	*p*_diff_
Happiness							
Medicated^a^	19	−0.164	−0.256	−0.073	<0.001	0	0.84
Unmedicated	3	−0.197	−0.502	+0.108	0.21	0	
Sadness							
Medicated^a^	18	−0.106	−0.260	+0.048	0.18	56	0.65
Unmedicated	3	+0.026	−0.515	+0.568	0.92	63	

CI, Confidence interval.

a Studies classified as ‘medicated’ include those where only a proportion of participants were medicated (Gur *et al.* 1992; Kan *et al.* 2004; Bediou *et al.* 2005; Mendlewicz *et al.* 2005; Joormann & Gotlib, 2006; Langenecker *et al.* 2007; Wright *et al.* 2009; Douglas & Porter, 2010; Milders *et al.* 2010; Anderson *et al.* 2011; Arteche *et al.* 2011; Derntl *et al.* 2012; Vederman *et al.* 2012).

### Small study bias

There was evidence of small study bias for the combined analysis (*p* = 0.003), while for the stratified analyses this was indicated for sadness (*p* = 0.028) and anger (*p* = 0.003). Adjusting for possible publication bias against null results using Duval and Tweedie's trim and fill method (Duval & Tweedie, 2000) indicated a reduced effect-size estimate in the combined analysis (*g* = −0.08, 95% CI −0.18 to +0.01), and the sadness (*g* = +0.04, 95% CI −0.12 to +0.20) and anger (*g* = 0.01, 95% CI −0.18 to +0.16) stratified analyses.

### Power analysis

The effect-size estimate indicated by our combined meta-analysis (*g* = −0.16) suggests that a sample size of approximately 615 cases and 615 controls would be required to detect a deficit in ER with 80% power at an alpha level of 0.05. The median sample size among studies included in our meta-analysis was 21 cases and 25 controls, which would correspond to 8% power to detect an effect size of this magnitude.

## Discussion

Our findings indicate a general ER deficit associated with MDD. In addition, analyses stratified by emotion indicate that the recognition of sadness is uniquely preserved, while recognition of the other basic emotions is impaired. We also did not find any evidence that study-level characteristics modified these results, suggesting that these effects may be relatively robust to diagnostic criteria, task parameters and other design factors. Given the variability in these factors across studies, this finding was unexpected, and may suggest that despite the small effect size of the ER deficit, this is a robust feature of MDD.

Medication status among cases did not appear to modify the association of depression with ER. However, this analysis included only three studies where depressed patients were unmedicated at time of testing, making it difficult to draw firm conclusions on the effects of medication on ER in this population. Details of current psychological treatment, which may also modify ER, were often unreported and therefore could not be systematically examined. It is noteworthy that studies of medicated patients tended to report a greater deficit in the recognition of sadness than the studies including only unmedicated patients, although there was not sufficient statistical power to evaluate whether this was a consistent effect. Clearly, further research with untreated depressed samples is required in order to better understand how ER deficits are associated with MDD, rather than medication or therapy *per se*. In particular, a wide body of research suggests that antidepressant medication reduces the recognition of, and neural responses to, negative facial expressions in healthy participants and patients with MDD (see Pringle *et al.* 2013). In the relative absence of data from unmedicated patients, it is possible that the current results are a marker of medication status as opposed to the disorder itself. Nonetheless, the results of our analyses stratified by emotion suggests that there is no unique relationship between MDD and the accurate recognition of happiness, as previously proposed (Gur *et al.* 1992; Bourke *et al.* 2010). Instead, the impaired recognition of happiness is merely part of a general recognition deficit across the other basic emotions. Response bias (i.e. the tendency to label ambiguous faces as positive *v.* negative) was not systematically reported in these studies and therefore has not been directly compared.

Contemporary theories of depression emphasize the importance of negative biases in ER as an important causal factor in illness etiology (Disner *et al.* 2011; Roiser *et al.* 2012). In particular, attentional, perceptual and interpretative biases towards negative material is believed to fuel negative self-referent schema in depression (Roiser *et al.* 2012). The current results are broadly consistent with this framework, since the recognition of sadness was preserved across a general landscape of ER deficits in depression. In other words, the recognition of sadness may be greater in relative terms, compared to the other emotional inputs (including happiness). However, the current results are not consistent with a more general negativity bias in depression in terms of accuracy of facial expression recognition. Based on the findings of a recent study, a negative bias in the interpretation of neutral faces rather than accuracy deficits in ER may represent a vulnerability factor for major depression in at-risk individuals (Maniglio *et al.* 2014). Given the effects of medication on the detection of negative emotion in facial expressions (Harmer *et al.* 2004, 2006), this conclusion needs to be qualified by noting the scarcity of research investigating ER in unmedicated patients. Future research should prioritize assessing ER (and associated measures) in patients free of medication. It is also worth noting that psychological treatments may also impact the processing of emotion in facial expressions, which indicates that studies in patients who are receiving neither pharmacological nor psychological treatments may be informative.

While our results indicate that MDD is associated with a general deficit in ER, the size of this effect is small. One consequence of this is the low statistical power of individual studies in our meta-analysis to detect effects of these associations, with the largest study achieving only 34% power to detect the effect size indicated by our meta-analysis. The problems associated with low statistical power have recently been described, and include an increased likelihood that a statistically significant finding reflects a false positive (Ioannidis, 2005; Button *et al.* 2013*b*). Rather than being endemic to a particular domain, the problem of low statistical power appears to be pervasive across several fields in the biomedical sciences. Our results therefore indicate the need for studies of ER deficits on a scale far larger than has been achieved to date. New technologies and data collection methods, such as the use of Internet and smartphone platforms, could help achieve this (Mar *et al.* 2013), and recent studies have shown that data collected via the Mechanical Turk are of comparable fidelity to those collected in a traditional laboratory setting (Crump *et al.* 2013). However, one important limitation of this approach is that it may be difficult to obtain data on clinical status except via self-report.

Our positive test for small study bias reveals evidence of possible publication bias against null results in this literature. This arises when researchers decide to not submit negative findings for publication, largely due to the prevailing tendency for journals to reject papers reporting null findings (Thornton & Lee, 2000). While we sought unpublished studies, as is common practice in conducting meta-analyses, we did not receive any responses. Given the presence of small study bias, we adjusted using Duval and Tweedie's trim-and-fill method (Duval & Tweedie, 2000) in order to account for small-study effects, where smaller studies in a meta-analysis tend to show larger treatment effects (Sterne *et al.* 2000). While reduced in strength, evidence of a general deficit in ER in depression remained.

There are a number of limitations to the present study which should be considered when interpreting these results. First, we excluded studies using stimulus sets generated using schematic or artistically rendered faces. This was done due to the lack of perceived ecological validity for schematic or artistically rendered faces, compared to human facial expression stimuli. We therefore cannot say whether our results would apply to tasks using schematic or artistically rendered faces. Second, there was insufficient data among studies included in our analysis to conduct meta-analysis on response bias. The investigation of false alarms from recognition tasks would offer further insight into the nature of the observed deficits, and would allow us to potentially identify biased responding for specific emotions, i.e. the tendency to mislabel ambiguous faces as sad or happy. However, there were minimal false alarm data available for analysis in the present study; future studies should report false alarm data consistently, alongside accuracy data. Third, we were limited in our ability to draw conclusions on the effect of medication status on ER in depressed individuals as few studies in our analysis assessed unmedicated cases. While the data available did not provide strong evidence that the recognition of emotion differs by medication status, contrary to our expectations given the literature on the effects of antidepressants on ER, future studies explicitly designed to test this (i.e. including both medicated and unmedicated cases) are required. Additionally, we were unable to investigate whether symptom severity moderated recognition performance as these data were not uniformly reported. As elevated depressive symptoms appear to predict poorer performance on recognition tasks (Kohler *et al.* 2011) accounting for symptom severity would be helpful when investigating the effect of medication status on performance. Finally, while we have determined a more accurate estimation of the size of the effect of depression on ER performance, it is not clear how these effects may translate to clinical significance. Therefore more research is needed to explore the relationship between ER and symptom severity.

In conclusion, our analyses confirm a general deficit of ER in depressed individuals compared to controls, albeit with a small effect size. Studies thus far have been considerably underpowered to detect this effect, and primary studies with much larger sample sizes will be required to properly investigate this association. Of the six basic emotions, only the recognition of sadness appears to be spared in depression, and there appears to be no specific association with impaired recognition of happiness. Further research comparing both medicated and unmedicated patients would offer new insights into the effects of depression on ER, with possible implications for treatment.

## Supplementary material

For supplementary material accompanying this paper visit http://dx.doi.org/10.1017/S0033291714002591. click here to view supplementary material
