# Supplementary material for: Meta-analysis of emotion recognition deficits in major depressive disorder
Source: Psychol Med. 2014 Nov 14;45(6):1135–44. doi: 10.1017/S0033291714002591 (PMC4712476; doi:10.1017/S0033291714002591)
Supplement: Supplementary file 1 [file S0033291714002591sup001.docx]

**SUPPLEMENTARY MATERIAL**

*Quality of Studies*

Table S1. *Assessment of quality of studies.*

| Study (year) |  | Selection | | | | | | |  | Comparability |  |  |  | Outcome | | |  | Total |
| --- | --- | --- | --- | --- | --- | --- | --- | --- | --- | --- | --- | --- | --- | --- | --- | --- | --- | --- |
|  |  | 1 |  | 2 |  | 3 |  | 4 |  | 5 |  | 6 |  | 7 |  | 8 |  |  |
| Anderson (2011) |  | ✫ |  | ✫ |  | ✫ |  | ✫ |  | ✫ |  | ✫ |  | ✫ |  | ✫ |  | 8 |
| Arteche (2011) |  | ✫ |  | ✫ |  | ✫ |  | ✫ |  | .. |  | .. |  | ✫ |  | ✫ |  | 6 |
| Bediou (2005) |  | ✫ |  | .. |  | ✫ |  | ✫ |  | .. |  | .. |  | ✫ |  | ✫ |  | 5 |
| Derntl (2012) |  | ✫ |  | ✫ |  | ✫ |  | ✫ |  | ✫ |  | ✫ |  | ✫ |  | ✫ |  | 8 |
| Douglas (2010) |  | ✫ |  | ✫ |  | ✫ |  | ✫ |  | .. |  | .. |  | ✫ |  | ✫ |  | 6 |
| Gaebel (1992) |  | ✫ |  | ✫ |  | ✫ |  | .. |  | .. |  | .. |  | ✫ |  | ✫ |  | 5 |
| Gollan (2010) |  | ✫ |  | ✫ |  | ✫ |  | ✫ |  | .. |  | .. |  | ✫ |  | ✫ |  | 6 |
| Gur (1992) |  | ✫ |  | ✫ |  | ✫ |  | ✫ |  | ✫ |  | .. |  | ✫ |  | ✫ |  | 7 |
| Joormann (2006) |  | ✫ |  | ✫ |  | ✫ |  | ✫ |  | .. |  | .. |  | ✫ |  | ✫ |  | 6 |
| Kan (2004) |  | ✫ |  | .. |  | .. |  | ✫ |  | .. |  | .. |  | ✫ |  | ✫ |  | 4 |
| Langenecker (2007) |  | ✫ |  | ✫ |  | ✫ |  | ✫ |  | ✫ |  | ✫ |  | ✫ |  | ✫ |  | 8 |
| Leppanen (2004) |  | ✫ |  | ✫ |  | ✫ |  | ✫ |  | ✫ |  | ✫ |  | ✫ |  | ✫ |  | 8 |
| Mah (2010) |  | ✫ |  | ✫ |  | ✫ |  | ✫ |  | .. |  | .. |  | ✫ |  | ✫ |  | 6 |
| Mendlewicz (2005) |  | ✫ |  | ✫ |  | ✫ |  | ✫ |  | ✫ |  | .. |  | ✫ |  | ✫ |  | 7 |
| Milders (2010) |  | ✫ |  | .. |  | ✫ |  | ✫ |  | ✫ |  | ✫ |  | ✫ |  | ✫ |  | 7 |
| Naranjo (2011) |  | ✫ |  | ✫ |  | ✫ |  | ✫ |  | ✫ |  | ✫ |  | ✫ |  | ✫ |  | 8 |
| Persad (1993) |  | ✫ |  | ✫ |  | ✫ |  | ✫ |  |  |  | ✫ |  | ✫ |  | ✫ |  | 7 |
| Schaefer (2010) |  | ✫ |  | ✫ |  | ✫ |  | ✫ |  | .. |  | .. |  | ✫ |  | ✫ |  | 6 |
| Schepman (2012) |  | ✫ |  | ✫ |  | ✫ |  | ✫ |  | ✫ |  | ✫ |  | ✫ |  | ✫ |  | 8 |
| Sprengelmeyer (2011) |  | ✫ |  | ✫ |  | .. |  | ✫ |  | .. |  | .. |  | ✫ |  | ✫ |  | 5 |
| Vederman (2012) |  | ✫ |  | .. |  | .. |  | .. |  | .. |  | .. |  | ✫ |  | ✫ |  | 3 |
| Wright (2009) |  | ✫ |  | .. |  | .. |  | ✫ |  | .. |  | .. |  | ✫ |  | ✫ |  | 4 |

Selection items 1 and 2 assessed the adequacy and representativeness of the cases respectively, while items 3 and 4 assessed the selection and definition of controls, respectively. Comparability items assessed whether the studies matched cases and controls for gender (item 5) and age (item 6). Outcome items 7 and 8 assessed the method used to ascertain outcomes of interest and whether the same method was used for both cases and controls, respectively. A star denotes that the study received a point for that item.

While Vederman et al. (2012) rated poorly on the scale, we found no evidence for a difference in our results when excluding this study from our analyses. Therefore we retained this study in our analyses.
